# Supplementary material for: The evaluation of the General Health Questionnaire (GHQ-12) reliability generalization: A meta-analysis
Source: PLoS One. 2024 Jul 17;19(7):e0304182. doi: 10.1371/journal.pone.0304182 (PMC11253975; doi:10.1371/journal.pone.0304182)
Supplement: S1 Table — (DOCX) [file pone.0304182.s001.docx]

**Supplementary Information**

**S1 Table.** Summary of COSMIN Risk of Bias (RB) Checklist Assessments

| Studies (Author Year) | Sample Size | Reliability | Validity | Responsiveness |
| --- | --- | --- | --- | --- |
| Barrigón et al. 2016 | 504 | Adequate | Very Good | Adequate |
| Rathore et al 2022 | 18070 | Adequate | Adequate | Adequate |
| Lee & Kim 2020 | 4270 | Adequate | Adequate | Adequate |
| Centofanti 2018 | 773 | Adequate | Adequate | Adequate |
| Elovanio et al 2020 | 870 | Adequate | Very Good | Adequate |
| Endsley et al. 2017 | 32,083 | Very Good | Very Good | Very Good |
| Liu et al 2022 | 3,020 | Adequate | Adequate | Adequate |
| Guan & Han 2019 | 1,085 | Very Good | Adequate | Adequate |
| Zhong et al 2022 | 996 | Very Good | Adequate | Adequate |
| Oliveira et al 2023 | 3477 | Adequate | Adequate | Adequate |
| Lanfe et al. 2022 | 300 | Adequate | Adequate | Adequate |
| Romppel et al. 2017 | 342 | Adequate | Adequate | Adequate |
| Namjoo et al. 2016 | 405 | Adequate | Adequate | Adequate |
| Alaminos-Torres et al. 2022 | 9692 | Very Good | Very Good | Very Good |
| Beaudreuil et al 2022 | 286 | Adequate | Adequate | Adequate |
| Qin, et al, 2018 | 676 | Adequate | Adequate | Adequate |
| Kashyap & Singh 2017 | 1763 | Adequate | Adequate | Adequate |
| Anjara et al. 2020 | 164 | Adequate | Adequate | Adequate |
| Hung et al. 2023 | 47 | Adequate | Doubtful | Adequate |
| Kuipers et al. 2019 | 432 | Adequate | Adequate | Adequate |
