# Supplementary figures and images for: The evaluation of the General Health Questionnaire (GHQ-12) reliability generalization: A meta-analysis
Source: PLoS One. 2024 Jul 17;19(7):e0304182. doi: 10.1371/journal.pone.0304182 (PMC11253975; doi:10.1371/journal.pone.0304182)

**Supplementary Information**

**S1 Fig.** QUADAS-2 Assessments for Included Studies


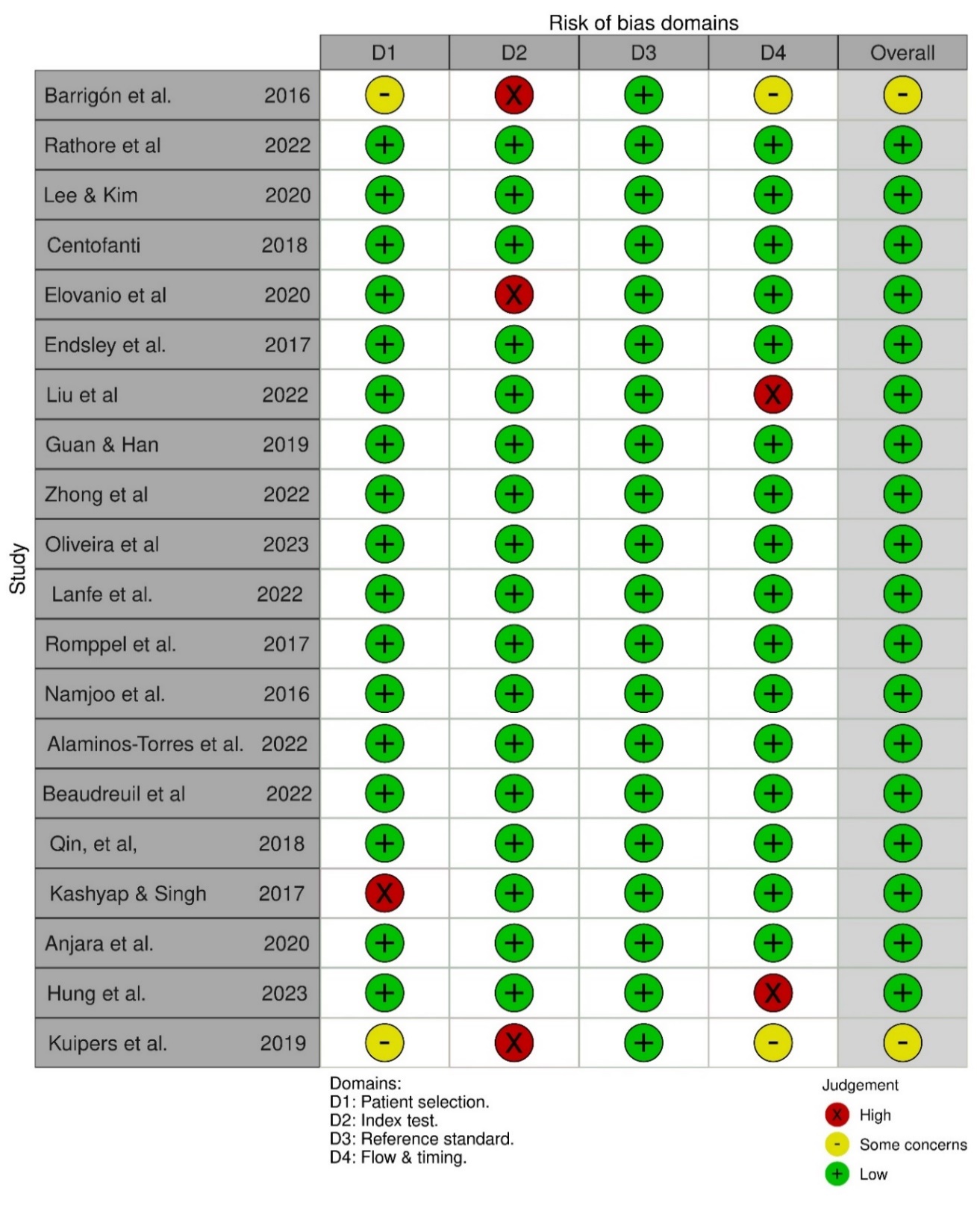

Supplement: S1 Fig — (DOCX) [file pone.0304182.s002.docx]
